# Should we Relax Abortion Reporting Requirements in Great Britain?

**DOI:** 10.1007/s10728-025-00512-7

**Published:** 2025-02-13

**Authors:** Jordan A. Parsons

**Affiliations:** Birmingham Medical School, University of Birmingham, Birmingham, UK

**Keywords:** Abortion, Autonomy, Confidentiality

## Abstract

In Great Britain, abortion has long proven to be contentious in the context of policy making, with it remaining a criminal offence. Despite progress over the last decade to permit home use of abortion medications and remote consultation, we have seen prosecutions in recent years. Regulatory frameworks such as this have been framed as ‘abortion exceptionalism’, such that termination of pregnancy is far more tightly regulated than comparable healthcare. One example of this exceptionalism is the strict abortion reporting requirements found in Great Britain. Per these requirements, any doctor providing abortion care must notify the relevant Chief Medical Officer or Public Health Scotland of each and every termination, including a startling amount of information about the patient. The extent of these requirements raises serious questions in relation to patient confidentiality and is, I suggest, an outlier in these terms. Further, it is questionable whether such reporting can be in any way said to be in the public interest. I begin by outlining the Abortion Regulations 1991, which apply in England and Wales, before considering the updated Scottish approach brought about by the Abortion (Scotland) Amendment Regulations 2021. I then move to examine the abortion reporting requirements against our general conception of patient confidentiality, highlighting the discordance. I ultimately argue that the requirements are not adequately justified and represent yet another, often forgotten, example of abortion exceptionalism in Great Britain. Thus, I suggest that all three nations that comprise Great Britain ought to further revise their approach to abortion data.

## Introduction

In Great Britain – and, indeed, globally – abortion has long proven to be contentious in the context of policy making [1]. Whilst on an individual level many people consider their view to be uncontroversial, in the reality of the policy arena this has not been the case. A piece of Victorian legislation – the Offences Against the Person Act 1861 – maintains termination of pregnancy as a criminal offence,^1^ punishable by custodial sentence. Despite progress over the last decade to permit home use of abortion medications and remote consultation [2, 3], we have seen prosecutions in recent years [4] – most notably that of Carla Foster.^2^ Regulatory frameworks such as this have been framed as ‘abortion exceptionalism’ [6, 7], such that termination of pregnancy is far more tightly regulated than comparable healthcare.

Such exceptionalism is pervasive, to the extent that even where abortion is accessible in Great Britain, it might only be considered a result of abortion-*permissive* legal frameworks rather than abortion-*supportive* [8]. Indeed, Lee notes how there being ‘no reference to freedoms and rights for women who want to terminate a pregnancy’ within the Abortion Act 1967 highlights how it cannot be considered liberal legislation [9]. This exceptionalism is not limited to higher-level questions such as criminalisation. It can be similarly observed in the finer details of abortion provision, such as the inclusion of a right of healthcare professionals to conscientious objection.^3^ Internationally, abortion exceptionalism can be seen in, for example, the United States’ so-called TRAP (targeted restrictions on abortion providers) laws [7, 10, 11].

My focus in this paper, however, is the strict abortion reporting requirements found in Great Britain.^4^ Per these requirements, any doctor providing abortion care must notify the relevant Chief Medical Officer of each and every termination. In England and Wales, this notification is to include a startling amount of information about the patient, with Scotland operating an alternative reporting process similar in extent. The extent of these requirements raises serious questions in relation to patient confidentiality and is, I suggest, an outlier in these terms. Further, it is questionable whether such reporting can be in any way said to be in the public interest even if this was central to the intention behind the reporting requirements.^5^

I begin by outlining the Abortion Regulations 1991, which apply in England and Wales, before considering the updated Scottish approach brought about by the Abortion (Scotland) Amendment Regulations 2021. I then move to examine the abortion reporting requirements against our general conception of patient confidentiality, highlighting the discordance. I ultimately argue that the requirements are not adequately justified and represent yet another, often forgotten, example of abortion exceptionalism in Great Britain. Thus, I suggest that all three nations that comprise Great Britain ought to further revise their approach to abortion data.

## Background to the Abortion Regulations 1991

In addition to the more widely recognised statutory grounds for abortion and two-doctor approval, the Abortion Act 1967 stipulated that regulations be made to require any registered medical practitioner terminating a pregnancy to ‘give notice of the termination and such other information relating to the termination as may be so prescribed’.^6^ This notice is to be given to the relevant Chief Medical Officer, depending on which nation the termination takes place in.^7^

In 1968, the required regulations were made in their original form. The Abortion Regulations 1968 extended to England and Wales, with Scotland separately making its Abortion (Scotland) Regulations 1968. Both sets of regulations were subject to various revisions over the proceeding years, before being replaced entirely in 1991 by the Abortion Regulations 1991 and Abortion (Scotland) Regulations 1991. I will examine Scotland’s approach shortly, so will for now focus on England and Wales.

Amendments to the original 1968 Regulations themselves tell a story of ever tighter regulation. Notably, those changes made following the 1974 Lane Report [13]. The Lane Report – or the Report of the Committee on the Working of the Abortion Act – compiled a wealth of evidence on practice surrounding the Abortion Act 1967 over a short initial period following its enactment. In doing so, it made a range of recommendations, several of which pertain to abortion reporting requirements.

Then Secretary of State for Social Services, Barbara Castle MP, responded to the report by initiating a ‘study of the procedures and forms set out in the regulations’ and ‘considering the whole question of the procedure for the certification and notification of abortions’.^8^ The changes that followed were significant and a marked increase in state oversight of abortion provision. In notifying the Chief Medical Officer, there was a new requirement for the two medical practitioners certifying the termination to confirm that they saw and/or examined the patient first.^9^ This was a clear attempt to address concerns that doctors were certifying patients as satisfying one of the statutory grounds without ever having met them. It also reinforces the Committee’s view that ‘it is in the interests of the patient as an individual that the abortion decision be taken by doctors’ (para 190) [13], which Temkin notes is an unexplained view [14], and one which characterises the consistent medicalisation of abortion. Further, as regards confidentiality in particular, an amendment allowed the Chief Medical Officer to disclose information collected through the notification process to be shared with the General Medical Council when requested ‘for the purpose of investigating whether there has been serious professional misconduct by a registered medical practitioner’.^10^ Again, seeking to increase oversight and, arguably, leave doctors worried about consequences even if they are acting in good faith.

Indeed, the general views of the Committee come through in the Report such that they were clearly not centring quality healthcare as a consideration. There is no clear medical benefit to reporting the patient’s date of birth to the Chief Medical Officer, but such a requirement is somewhat reinforced by the Committee’s apparent fixation on ‘special categories’ of people who may seek an abortion. Whilst the date of birth requirement was already in place, the Report expresses its highly paternalistic view of these age-based ‘special categories’ in an inconsistent manner. For example, empowering those under the age of 16 to make their own decisions on termination (para 240) [13] whilst aiming to change the minds of those over 35 as they are likely seeking an abortion because of ‘embarrassment’ (para 264) [13]. Another ‘special category’ is those who seek successive abortions, with their ‘brazen’ attitude supposedly indicating psychological problems (para 269) [13]. The Report goes as far as to raise sterilisation as a possible response to this concern (para 273) [13].

This attempt to articulate a typology of abortion seekers highlights the way in which the reporting requirements exert state control, both directly and indirectly. Tracking who accesses abortion care in such detail without a legitimate health-related justification allows for such dissection as a prime example of abortion exceptionalism, which contributes to the continuation of abortion as a topic of societal debate rather than a matter of individual healthcare.^11^ Indeed, this focus on constructing “types” of people who access abortion care, with an emphasis on weakness, vulnerability, and desperation, has remained pervasive [16].

Returning to the current iteration, the Abortion Regulations 1991 stipulate the precise information that is to be collected by any doctor providing an abortion to include in their notification. Schedule 2 provides the details as part of the notification form itself. More commonly known as the “yellow form”,^12^ the officially designated HSA4 form has quite extensive data requirements. Across the 6-page form, professionals are required to collect and report information pertaining to the abortion itself, including:

- Details of the doctor who performed the termination
- Details of the two doctors that authorised the termination^13^
- Place of termination
- Procedure used
- Date of admission, termination, and discharge (as applicable^14^)
- Gestation
- Grounds for termination
- Complications

In addition, the yellow form records extensive personal information about the patient, including:

- Name
- Address
- Date of birth
- Ethnicity
- Marital status
- Previous pregnancies

That final datapoint on previous pregnancies is especially detailed, requiring specific information on the number of livebirths or stillbirths over 24 weeks, spontaneous miscarriages or ectopic pregnancies, and legal terminations.

Whilst some of this personal information may be rather commonly collected by forms in a healthcare setting – such as name, address, and date of birth – other datapoints have a distinct social angle to them. Most notably, the field on marital status.

Initially, the Abortion Regulations 1991 required that a hard copy of this form was sent to the relevant Chief Medical Officer ‘in a sealed envelope within 7 days of the termination’.^15^ Understandable for the time, perhaps. Then, with amendment regulations in 2002, there was a modernisation of these requirements to allow for forms to be sent either in a sealed envelope or digitally and within an extended period of 14 days.^16^ There were no changes to the data required, however.

Compliance with the reporting requirements is a legal requirement. Failure to do so presents legal difficulties, opening doctors up to prosecution for a summary offence.^17^ That can include where a form is submitted mostly completed, but with one applicable field left empty. There is, then, absolutely no professional discretion afforded by the Regulations. A doctor cannot decide that in a given situation it is not appropriate for a particular piece of information to be reported. For example, if a patient does not wish to divulge their marital status.

I will shortly come to examine these requirements against ordinary understandings of confidentiality in healthcare. For now, it is at least worth acknowledging how unusual these reporting requirements are. Even with notifiable infectious diseases, which constitute a clear public health concern, doctors are afforded some discretion in precisely what information they report.^18^ The suggestion that there is a greater public health concern with the termination of pregnancy than with infectious diseases can only realistically be said to be rooted in a social justification, not a health justification.

## The Scottish Approach

I have intentionally contained my discussion to England and Wales thus far – despite the overall focus on Great Britain – because Scotland operates under slightly different regulations. There has been a similar history with the Abortion (Scotland) Regulations 1968 and various amendments thereafter, then culminating in repeal and replacement with the Abortion (Scotland) Regulations 1991. The changes discussed above in relation to England and Wales following the Lane Report were mirrored in Scotland per the Abortion (Scotland) Amendment Regulations 1976, so I will not reproduce them here. By 1991, both sets of regulations largely mirrored one another. However, changes after this point have seen them somewhat out of alignment ever since.

When the 2002 amendments were made in England and Wales, Scotland did not follow suit. For a while, then, Scotland maintained the far stricter requirement for notification by sealed envelope within seven days. Then, in December 2021, the Abortion (Scotland) Amendment Regulations 2021 were made, coming into force in May 2022. Amongst various changes to the 1991 Regulations was a near realignment with England and Wales by way of extending the notification period. Being slightly more generous than the 2002 amendments, Scotland’s new requirement is for notification to the Chief Medical Officer to take place ‘before the fifteenth day of the calendar month immediately following the calendar month in which the practitioner terminated the pregnancy’.^19^ Another amendment brought Scotland closer to England and Wales in permitting electronic notification^20^ – something of undoubtedly greater importance following the introduction of telemedical abortion care pathways [3].

One further amendment, however, is most important. In a rather more drastic change, the extent of data required in a notification was reduced to now include only the name of the practitioner and their employer.^21^ That is, the extensive details about the patient’s healthcare and personal life no longer have to be reported to the Chief Medical Officer in Scotland. This is something that was not done by the 2002 amendments in England and Wales, meaning the two sets of regulations are now starkly different on this aspect.

That said, whilst the Regulations themselves may no longer require this reporting in Scotland, similarly detailed reporting continues through a different avenue. It was made clear by the Scottish Government in instigating the amendments that the purpose was ‘to modernise the process for providing notifications to the [Chief Medical Officer] and also streamline the process by which data about abortions in submitted by providers’ [17]. No mention was made of seeking to alter the underlying goal of collecting such data. Rather, it was about process and simplification for the sake of professionals. This was further clarified in the Policy Note published alongside the Amendment Regulations, which makes clear the intention that Public Health Scotland will continue to collect abortion data, albeit with the specific range of data to be determined [18]. The key change, then, is that these data are now sent directly to Public Health Scotland by abortion providers through the Termination of Pregnancy Submissions Scotland (ToPSS) system – the office of the Chief Medical Officer no longer acts as a middleman.

Subsequent reports by Public Health Scotland demonstrate some changes to the datapoints now used. For example, providers no longer have to report the names of the two practitioners, the dates of admission or discharge, or the marital status of the patient. However, additional datapoints have been added – most notably, the patient’s ethnicity [19]. The result being that, arguably, Scottish reporting now involves *more* personal information about those accessing abortion services than in England and Wales.

With the amendments, however, comes the loss of statutory power behind reporting. Direct reporting to Public Health Scotland allows for some discretion, unlike the previous yellow form that had to be completed in its entirety. For example, the latest report shows that the additional datapoint of ethnicity was reported as ‘refused, not provided, or not known’ in 9.9% of terminations in the year 2023 [20]. Whilst there is not currently data on the nature of conversations taking place to collate this information,^22^ it is clear that at least some patients are being given the option not to provide full details without this impacting on their access to abortion care – something that is not the case in England and Wales.

Overall, there are aspects of the 2022 changes in Scotland that reduced the reporting burden on both professionals and patients, but others that increased it again. Reporting in Scotland remains, then, a clear example of abortion exceptionalism – just without the same statutory footing.

## An Outlier in Confidentiality Terms?

Where the sort of details recorded by the “yellow form” and ToPSS system are being collected and shared as part of a healthcare encounter in Great Britain, there is an inevitable question of patient confidentiality. It is entirely understandable that some may not want that information shared beyond the healthcare team providing their care. Some may also prefer not to tell their healthcare team all those details, particularly as several of the datapoints have no clinical relevance. For example, whether a patient is single, married, divorced, etc. should not make any difference to how a healthcare professional responds to a request to terminate a pregnancy – the loss of that particular datapoint in Scotland being a welcome change.

That this information is collected and shared regardless of the patient’s consent does not necessarily present a problem. There are reasons why patient confidentiality might be legitimately broken. However, the bar for such legitimacy is high, and it is not clear that it is met in the case of these abortion reporting requirements. We must question, then, whether it is appropriate that further amendments are made to abortion reporting in Great Britain.

### Patient Confidentiality

Confidentiality can easily be recognised as playing an important role in society in general. In the UK, this importance has been firmly established both through the common law duty of confidentiality^23^ and with more recent statutory affirmations.^24^ In the healthcare context, the importance is greater, for it is ‘critically important to the effective functioning of trusting therapeutic relationships’ [21]. Hence it featuring prominently in professional guidance from, for example, the General Medical Council [22] and British Medical Association [23], including advising professionals on very specific matters [24].

Despite its importance, it is simultaneously recognised that there are and must be limits to patient confidentiality [25].^25^ Most obviously is where healthcare professionals share information about a patient for the purposes of providing care. Few would regard this as a “breach” of confidentiality as there is thought to be a reasonable expectation that such information sharing is necessary – hence Stanton’s explanation of the ‘surprise test’ as a means of professionals considering when explicit consent might be appropriate [26].^26^ There are also instances where a patient might themselves consent to the wider sharing of their health information, such as for the purposes of research. Again, few would consider this a “breach” of confidentiality, assuming the consent provided by the patient is valid.

Then we come to the more challenging instances of sharing a patient’s health information; those where the information is shared *without* the patient’s consent and are thus very clearly breaches of confidentiality. First is where a breach is expressly required by law. There are many laws which stipulate that a healthcare professional must disclose certain information, ranging from the more obvious Health Protection (Notification) Regulations 2010 with regard to notifiable infectious diseases to the Terrorism Act 2000’s requirement to share any information that may prevent terrorism.^27^ These are, in one sense, rather straightforward. Even if the healthcare professional may feel uncomfortable, or the patient protests, there is a clear legal basis for breaking confidentiality such that the healthcare professional has no choice.

Alternatively, confidentiality might be breached in the public interest. To satisfy this public interest threshold, the consequences of *not* breaching confidentiality must be serious. For example, the prevention of serious harm or a criminal act. To balance this against the importance of maintaining confidentiality, the bar is necessarily high. Nonetheless, both sides of the coin have a clear consequentialist basis.

The public interest justification is more subjective and affords the healthcare professional a level of discretion that is absent where disclosure is required by law. However, it is important to recognise that breaches required by law are also breaches in the public interest. Most instances where the law requires the sharing of a patient’s health information are justified on the basis of preventing harm and/or crime in some way. The difference being that some have been codified, thus removing the need for any healthcare professional to weigh up the public interest before disclosing. But the underlying justification for setting aside absolute confidentiality is the same.

There are various further complexities depending on specific circumstances, such as where a patient lacks capacity, but these are not my focus.^28^ My overview of confidentiality here is intentionally brief as it serves only to highlight the centrality of the public interest to instances where confidentiality might be breached by a healthcare professional – regardless of whether such a breach is expressly stipulated by law.

#### General Data Protection Regulation

It is worth briefly touching on the relevance of the General Data Protection Regulation (GDPR). In 2018, GDPR came into force throughout the European Union. It remains in force in a post-Brexit UK per the European Union (Withdrawal) Act 2018, now referred to as UK GDPR.^29^ GDPR seeks to protect the interests of individuals by stipulating requirements for the processing of personal data.

Recital 26 makes clear that GDPR does not apply to anonymous data,^30^ which is defined as ‘information which does not relate to an identified or identifiable natural person or to personal data rendered anonymous in such a manner that the data subject is not or no longer identifiable’.^31^ Thus, once data collected in line with the Abortion Regulations 1991 and the Abortion (Scotland) Regulations 1991 is processed and compiled for the purposes of publishing abortion statistics, GDPR is no longer applicable. However, in initial handling it undeniably is applicable as the notification forms include the name, address, and date of birth of the patient (in addition to other information that might enable identification). GDPR, then, at least has relevance to abortion reporting at some point in the process. That raises the question as to the alignment of both sets of regulations with GDPR.

Several articles are of relevance here, potentially providing a basis for the reporting requirements. Perhaps unsurprisingly, they do not diverge significantly from the general principles of confidentiality detailed above. First, GDPR does not apply where competent authorities process data to prevent, investigate, detect, or prosecute a criminal offence.^32^ Abortion provided outside of the provisions of the Abortion Act 1967 constitutes a criminal offence, so this is potentially engaged. It would come down to whether the reporting requirements in any way contribute to such crime prevention efforts, which I will discuss shortly. It must also be recognised that GDPR permits the processing of data where the controller (in this case, the abortion provider) is doing so in compliance with a legal obligation.^33^ Thus, the force of the Abortion Regulations 1991 and Abortion (Scotland) Regulations 1991 carries regardless of whether its bases align with GDPR more generally. Processing would similarly be lawful on the basis of the patient consenting to it.^34^ Though this would require demonstrable consent to data processing, which is not a stipulation of either set of regulations even if it might be said to be good practice.

Also of relevance is Article 9 given health data’s recognition as a special category.^35^ Such data can be processed in line with GDPR for one of several reasons, three of which can be said to apply here. First, for ‘reasons of substantial public interest’ in line with domestic law that is ‘proportionate to the aim pursued’.^36^ Second, for the management of health and social care systems and services on the basis of domestic law.^37^ Third, for archiving or statistical purposes in the public interest.^38^ These speak to the public interest justification applicable to the handling of confidential data more generally, which will be considered shortly.

Whatever the justification, GDPR does require data minimisation. That is, only what is required for the intended purposes should be collected.^39^ In the case of abortion reporting requirements, there is no explicitly stated purpose within either the Abortion Act 1967 or either set of regulations. However, we can infer that the purpose is for the monitoring of care provision given that the primary use of all collected data is the production of statistical reports. This makes it difficult to assess whether specific datapoints can be said to be required – if the purpose is to report these data, they are necessarily required, even though one might argue the purpose itself is questionable.

### Can Abortion Reporting Requirements be Justified?

Given this understanding of confidentiality and when breaches are considered justified, the question remains as to where the abortion reporting requirements fit. Of course, that the regulations stipulate this breach of confidentiality means that it is lawful – more so, it is legally required.^40^ My contention, however, is that the regulations and associated reporting processes themselves are ethically unjustified; they are out of step with our broader understanding of confidentiality in healthcare and should not be excused simply because parliamentary sovereignty supersedes this. Taking the recognised justification of public interest, I suggest that there cannot be said to be a public interest in the collection of such detailed data on abortion care. As such, there is a need to consider whether there is a public interest in abortion reporting on the basis of either the prevention of harm or the prevention of crime, for a lack of public interest would demonstrate a lack of justification for the regulations.

#### Prevention of Harm or Crime

To first consider harm, it is unclear what harm could be said to be caused by *not* reporting an abortion. This particular framing is important as the focus needs to be on the harms caused by *maintaining* confidentiality to consider whether a breach is justified. The burden of proof is very much on the side of breaching with confidentiality as the default – confidential until proven otherwise.

Arguments relating to the harms caused by abortion are ordinarily directed at the procedure itself, in that it harms the embryo or fetus which, for some, has personhood. Whilst I do not find such arguments convincing anyway, even if one were to endorse them, they do not extend to justifying abortion reporting on the basis of this harm. Those opposed to abortion on such grounds would see the harm as having already happened by the abortion being performed. At most, this argument might inch towards reporting the abortion as a crime, but provided it was lawfully provided in line with the Abortion Act 1967 this would not carry weight (the question of crime prevention will be revisited shortly).

There are, then, no obvious harms that might arise where an abortion is not reported in line with the regulatory requirements. That brings us to the question of preventing crime. Of course, abortion remains a criminal offence in Great Britain, so it is feasible that an abortion that is taking place is unlawful. However, it is not a realistic expectation that data reported in line with the regulations would assist with the prevention of such crime – even if one of the intentions behind the reporting requirements was to reduce the number of unlawful abortions.^41^ Similarly to the question of harm, at the point of reporting an abortion, that abortion has already taken place. Even if it later transpired that it had been provided unlawfully – and even that is unlikely to be ascertainable from the yellow form itself – that abortion will already have taken place so cannot be prevented. Experience since the introduction of reporting requirements clearly demonstrates that they have not impacted on the prevention of crime in this way. The data collected are not managed by the police and, following a case in the early 2000s (which will be discussed shortly), reporting of the data does not allow for the identification of potentially unlawful instances of termination.

Further, their willingness to comply with the reporting requirements in any given situation suggests that the relevant doctor is confident that the care they are providing is in line with the Abortion Act 1967. In the frankly unlikely scenario that a doctor is intentionally providing abortion care unlawfully, one would imagine that doctor would marry that with a decision not to report any such abortions to the Chief Medical Officer (or Public Health Scotland). Otherwise they would be confessing to a crime.

There is also the related (though separate) question of a healthcare professional breaching confidentiality to report a suspected unlawful attempt by a patient to terminate their pregnancy. That is, where the abortion does not take place as the doctor does not consider the circumstances reconcilable with the Abortion Act 1967. However, this is the subject of specific confidentiality guidance from the British Medical Association [27]. It is thus distinct from the abortion reporting requirements themselves which solely concern terminations that are carried out rather than more general care relating to abortion, further clarifying how the requirements fail to align with our ordinary justification(s) for breaching confidentiality.

It appears, then, that abortion reporting acts to prevent neither harm nor crime. As such, our ordinary understanding of the public interest as a justification for breaching confidentiality is not applicable, suggesting the reporting not to be appropriate. Let us consider, then, what else might be thought of as justifying such a breach.

#### Reporting as Abortion Exceptionalism

The abortion reporting requirements can only sincerely be said to be for reasons of data collection and monitoring – that is, exerting control. Indeed, all data collected are analysed for the purposes of producing annual reports on abortion care [20, 28].^42^ Drawing on the extensive data collected, these reports chart changes in abortion care over the years in a huge amount of detail. Whilst they may provide a hugely useful resource for some – researchers working on the topic of abortion care in particular – the existence of such detailed reports cannot be said to be in any way serving the public interest to the extent that breaching confidentiality is justified. If utility to researchers were considered sufficient justification, then similarly extensive reporting would be justified for each and every medical procedure carried out in this country.

Even for monitoring care provision, if one sought to defend reporting for such purposes, there is inadequate justification for the precise nature of collected data. Whilst I would still consider it relatively weak, one could at least feasibly make the case for collecting some of the information relating specifically to the procedure. For example, enabling an understanding of the average admission for surgical abortion or the balance of different abortion methods used, as such information *could* be used to improve services.^43^ Personal details, however, are not necessary for such purposes. There is no reason why the marital status of those accessing abortion care would need to be understood as there is no realistic way in which such information might be harnessed to improve care – the same options of abortion care, as well as any requested advice on reproductive health, should be available equally to all patients regardless of marital status.

Reporting the patient’s address is similarly to provide an unnecessary level of detail. If, for service improvement purposes, it is considered useful to understand the demands on abortion services in different locations, this could be captured in more general terms. For example, reporting a town or even first half of postal code. An understanding of the distribution of care need is never required to the extent of individual streets as, once again, there is no meaningful way in which the healthcare system can respond to improve care on a street-by-street basis.

Even if one is of the view that having some of these data is beneficial for service improvement purposes, it must simultaneously be acknowledged that in no other area of healthcare are data collected to this extent. Abortion care is unique in having such extensive data collected and made (anonymously) publicly available in annual reports. Almost all areas of healthcare provision could benefit from a more detailed understanding of service needs, responding to improve equity in provision, but that is not used to justify a legal requirement to set aside confidentiality and report this level of information to the Chief Medical Officer (or Public Health Scotland). The only feasible conclusion is that the reporting requirements in abortion care constitute yet another example of abortion exceptionalism.

Essentially, the regulations put forward the position that in all instances of abortion there is an appropriate justification for setting aside the importance of patient confidentiality. That there is something inherent in abortion care that presents a public interest in there being data available in an excruciating level of detail. The undertone here relates to abortion remaining a criminal offence in Great Britain and those seeking such care being characterised as necessarily vulnerable [16]. That is, the monitoring of abortion care to this extent is a matter of preventing crime, hoping that potential perpetrators will be deterred by being held accountable in this way. Though, as highlighted above, the ability of the reporting requirements to contribute to any such efforts is limited at best.

#### Justification Against Reporting

Beyond the passive lack of justification for having the reporting requirements, there is simultaneously active justification for *not* having them. The reporting requirements themselves have the very real potential to cause harm. First, they might deter some very vulnerable patients from accessing care. Consider, for example, a patient who is seeking to terminate a pregnancy they have kept secret for fear of being judged by members of their community. This individual might fear that the reporting is not perfectly confidential and thus be reluctant to provide such detailed information. Victims of abuse, too, may worry about their abuser finding out. Even someone who is seeking a termination with the support of their partner may be reluctant to provide details of past pregnancies if, for example, their partner is unaware of a past abortion. Such concerns were explicitly acknowledged during the passage of the Abortion Act 1967.^44^

It might be said that such concerns over information not being entirely confidential are, accounting for history, entirely reasonable. The Metropolitan Police were asked to investigate a late abortion for the reason of cleft lip/palate detailed in the 2001 abortion statistics – it being the only one (see Table 23 in the report) [29]. When a press release indicated West Mercia Police would investigate due to the location of the case, the press were able to identify the hospital and doctor concerned, resulting in anti-abortion activists campaigning against the doctor.^45^ Whilst the patient was not identified, it is reasonable for patients to be concerned about the possibility of their identification if people are able to identify the hospital and doctor – not to mention the concern over the harm to individual doctors where this happens.

This case was mentioned in a 2008/9 Information Tribunal wherein the Pro-Life Alliance sought the disclosure of more detailed information on abortions provided on the grounds of fetal abnormality. Commendably seeking to minimise the risk of individuals being identified – in response to a review following the abovementioned case investigated by West Mercia Police^46^ – the Department of Health^47^ had stopped reporting exact values of sensitive datapoints where they were below 10.^48^ The Tribunal ultimately found that Article 8 of the European Convention on Human Rights was not engaged (as had been the Department of Health’s argument). Nonetheless, it went on to note that if Article 8 were engaged, the Tribunal was still satisfied that such an interference was justified in accordance with the law as well as being proportionate and necessary for the prevention of crime as abortion is a criminal offence.^49^ Very explicitly, then, the Tribunal highlighted the prevention of crime as the justification for a breach of confidentiality should it arise in such circumstances.^50^

Regardless of the reason, when someone chooses to disengage with an abortion care provider on finding out about the reporting requirements there arises a risk that they will seek clandestine means of termination instead. This risks compromising patient safety. Wider discourse on the importance of patient confidentiality acknowledges the risk of disengagement where it is breached, but this is seemingly overlooked in the abortion context.

Second, even if the patient is willing to provide the information, asking them to do so may, in some cases, be traumatic. Here I am particularly talking about the information on previous pregnancies. This would require a patient to revisit past experiences of miscarriage and stillbirth for the sake of the form, or even past terminations which may have taken place for uncomfortable reasons. Whilst there may sometimes be a need for a healthcare professional to ascertain some of this information for medical reasons, a history to the extent required by the yellow form is ultimately unnecessary. It risks distressing patients for no justifiable reason.

As well as patients, the legal requirement to report such extensive information may cause healthcare professionals moral distress. Unlike instances where healthcare professionals might decide to breach confidentiality in the public interest, they have no choice but to report any termination they carry out. Some may struggle with this, feeling it is unjustified for the reasons I have outlined. Particularly if they face a patient who chooses to decline an abortion as they do not want their details reported, that professional may fear that the patient will seek to terminate their pregnancy by other, potentially dangerous, means. This lack of professional discretion could thus be harmful to abortion care providers, and further empirical research on this would be a welcome addition to the literature.

#### Summary

It is thus demonstrable that the nature and extent of the reporting requirements stipulated by the Abortion Regulations 1991 – and similarly actioned by Public Health Scotland – is not only unjustifiable, but effectively renders abortion care an outlier in confidentiality terms. Continued monitoring of abortion no doubt speaks to the long-term concerns of many in the policy sphere that the Abortion Act 1967 is being abused. Spanning from the early years, through the significance of the Lane Report [13], to more recent attempts to incorporate stipulations for ‘independent’ counselling,^51^ there have always been those in positions of power that have sought to tighten the state’s grip on abortion provision under the heading of safeguarding (even if some may question the motivation of some who are simultaneously vocally opposed to abortion [31]). Monitoring provision through reporting requirements might be thought to discourage practices that in any way diverge from the stipulations of the legislation. However, whether this is a realistic or, indeed, justifiable approach is, as I have shown, open to legitimate questioning.

Whilst not my focus here, it is worth acknowledging that my exploration of the problems with abortion reporting requirements may be similarly applicable in other jurisdictions. In the United States, for example, the Center for Disease Control^52^ began to compile abortion statistics from all states in 1969, at least initially under the guise of monitoring for safety.^53^ The so-called ‘Abortion Surveillance’ reports are similarly comprehensive, capturing information on the patient’s age, race, place of residence, marital status, gestation, abortion type, and reason(s) for termination [32].^54^ This reporting continues to this day [33].^55^ Even if the applicable legal frameworks vary, similar arguments against such detailed data collection and reporting on abortion care might reasonably apply in the United States as in Great Britain – and, indeed, other jurisdictions.

## Conclusion

My conclusion is tripartite. First, it is clearly demonstrable that the abortion reporting regulations in Great Britain are yet another example of abortion exceptionalism, and an example that is not commonly acknowledged. Second, that the reporting requirements imposed on abortion care providers are not justified in the public interest and are thus out of step with the realm of medical confidentiality more broadly. Third, based on these first two points, there is a clear need for abortion reporting regulations to be revisited.

The past decade has seen significant progress in the easing of overregulation in abortion care in Great Britain. Barriers to access have been removed by, initially, the introduction of home use of misoprostol, then further by remote consultation and medical abortion by post. The criminalisation of abortion care remains, which is itself a matter of significant debate, but there has at least been an improvement in abortion regulation in a practical sense if less so in a principled sense [7, 8]. A relaxation, or even total removal, of abortion reporting requirements is a natural next step in the gradual erosion of unjustified abortion exceptionalism. This will go some way to overcoming the fudge that is abortion policy in Great Britain, whereby it seeks to appease two conflicting positions. So, in response to the title question: yes. Yes, we absolutely should relax abortion reporting requirements in Great Britain.

## Footnotes

1 This Act does not extend to Scotland, but abortion is nonetheless a criminal offence in Scotland by virtue of common law. See sch.5, part.II, s.J1 Explanatory Notes to the Scotland Act 1998.

2 *R v Foster* [2023] EWCA Crim 1196. See also [5].

3 S.4 Abortion Act 1967.

4 This heavy approach to regulation through reporting requirements is by no means unique to Great Britain. Similar requirements can be found elsewhere, such as the United States. See, for example, [12].

5 HC Deb 13 July 1967, vol 750, col 1242, per Bernard Braine MP; HC Deb 13 July 1967, vol 750, col 1249, per David Steel MP.

6 S.2(1)(b) Abortion Act 1967.

7 S.2(2) Abortion Act 1967.

8 HC Deb 21 October 1975, vol 898, col 245.

9 Sch.2 The Abortion Regulations 1968, as amended by reg.5 of The Abortion (Amendment) Regulations 1976.

10 Reg.5(h) of The Abortion Regulations 1968 as amended by reg.3 of The Abortion (Amendment) Regulations 1976.

11 A comprehensive account of the history of the Abortion Act 1967 and many of the events that highlight its controversies can be found in the work of Sheldon and colleagues [1, 15].

12 No prizes for guessing why.

13 There is no requirement that the termination be performed by one of the authorising doctors.

14 Admission will not ordinarily be required for a medical abortion, for example.

15 Reg.4(1)(b) Abortion Regulations 1991 [as enacted].

16 Reg.4 Abortion Regulations 1991, as amended by reg.4(a)(ii) Abortion (Amendment) (England) Regulations 2002 and reg.4(a)(ii) Abortion (Amendment) (Wales) Regulations 2002.

17 S.2(3) Abortion Act 1967.

18 Reg.2(2)(f)-(g) The Health Protection (Notification) Regulations 2010.

19 Reg.4(1)(3) Abortion (Scotland) Regulations 1991, as amended by reg.3 Abortion (Scotland) Amendment Regulations 2021.

20 Whilst electronic notification is not explicitly stipulated in the Abortion (Scotland) Amendment Regulations 2021, regulation 4 of the Abortion (Scotland) Regulations 1991 is entirely substituted and no longer includes a sealed envelope requirement, thus having the effect of permitting electronic notification.

21 Reg.4(2) Abortion (Scotland) Regulations 1991, as amended by reg.3 Abortion (Scotland) Amendment Regulations 2021.

22 Certainly, empirical data on how these conversations may have changed since the amendments were made would be hugely useful in understanding the real impact.

23 *Attorney General v Guardian Newspapers (No. 2)* [1990] AC 109.

24 For example, the Human Rights Act 1998 and Data Protection Act 2018.

25 See also *W v Egdell* [1990] 1 All ER 835.

26 This test is a recommendation of the General Medical Council’s guidance [22].

27 S.38B Terrorism Act 2000.

28 A useful overview is provided by Snelling and Quick [21].

29 Reg.2 The Data Protection, Privacy and Electronic Communications (Amendments etc.) (EU Exit) Regulations 2019.

30 Recital 26(6) General Data Protection Regulation 2016.

31 Recital 26(5) General Data Protection Regulation 2016.

32 Art. 2(2)(d) General Data Protection Regulation 2016.

33 Art. 6(1)(c) General Data Protection Regulation 2016.

34 Art. 6(1)(a) General Data Protection Regulation 2016.

35 Art. 9(1) General Data Protection Regulation 2016.

36 Art. 9(2)(g) General Data Protection Regulation 2016.

37 Art. 9(2)(h) General Data Protection Regulation 2016.

38 Art. 9(2)(j) General Data Protection Regulation 2016.

39 Art. 5(1)(c) General Data Protection Regulation 2016.

40 Whilst Scotland no longer stipulates datapoints as in England and Wales, the Amendment Regulations reaffirm the role of Public Health Scotland in collating and reporting such data.

41 HC Deb 13 July 1967, vol 750, col 1242–1245, per Bernard Braine MP.

42 The report cited covers care provided in England and Wales. Similar reporting takes place in Scotland, by Public Health Scotland.

43 I stress could because the data are not used for a process of service improvement – not least because abortion services are predominantly delivered by contracted providers rather than the National Health Service. Those providers, it is worth noting, being charitable organisations, form a ‘highly distinctive and significant aspect’ of the history of abortion provision in Great Britain [15].

44 HC Deb 13 July 1967, vol 750, col 1243, per Bernard Braine MP.

45 *In the Matter of an Appeal to the Information Tribunal under Sect. 57 of the Freedom of Information Act 2000* (Information Tribunal Appeal No EA 2008/0074), at paras 18–19.

46 Ibid, at para 20.

47 Now Department of Health and Social Care.

48 Ibid, at para 27.

49 Ibid, at para 72.

50 For further discussion of this tribunal and its implications for confidentiality in healthcare more broadly, see [30].

51 HC Deb 7 September 2011, vol 532, cols 365, per Nadine Dorries MP.

52 Now Centers for Disease Control and Prevention.

53 Though it should be noted that states are not required to report data for inclusion in national reporting, so it is not necessarily as comprehensive as what is produced in Great Britain.

54 I cite here the 1972 annual summary. It acknowledges that there are previously published reports, which I reasonable presume start in 1970. However, the 1972 reporting is the earliest available on CDC Stacks at the time of writing.

55 For further history of abortion reporting in the United States, see [34].
